# Early-Life Nutritional Programming of Type 2 Diabetes: Experimental and Quasi-Experimental Evidence

**DOI:** 10.3390/nu9030236

**Published:** 2017-03-05

**Authors:** Alexander M. Vaiserman

**Affiliations:** D.F. Chebotarev Institute of Gerontology, Kiev 04114, NAMS, Ukraine; vaiserman@geront.kiev.ua; Tel.: +38-044-431-0558

**Keywords:** type 2 diabetes, famine, natural experiment, quasi-experimental design, epigenetics

## Abstract

Consistent evidence from both experimental and human studies suggest that inadequate nutrition in early life can contribute to risk of developing metabolic disorders including type 2 diabetes (T2D) in adult life. In human populations, most findings supporting a causative relationship between early-life malnutrition and subsequent risk of T2D were obtained from quasi-experimental studies (‘natural experiments’). Prenatal and/or early postnatal exposures to famine were demonstrated to be associated with higher risk of T2D in many cohorts around the world. Recent studies have highlighted the importance of epigenetic regulation of gene expression as a possible major contributor to the link between the early-life famine exposure and T2D in adulthood. Findings from these studies suggest that prenatal exposure to the famine may result in induction of persistent epigenetic changes that have adaptive significance in postnatal development but can predispose to metabolic disorders including T2D at the late stages of life. In this review, quasi-experimental data on the developmental programming of T2D are summarized and recent research findings on changes in DNA methylation that mediate these effects are discussed.

## 1. Introduction

Type 2 diabetes (T2D) is one of most common chronic diseases, constituting a serious social and economic problem in modern societies, both developed and developing. It is caused by insulin resistance resulting from decreased activity and enhanced obesity levels that occur with increasing age. T2D is considered to be adult-onset disease, since it typically occurred in middle-age and old adults. Generally, T2D occurs after the age of 40, although it is now increasingly diagnosed in younger patients [1]. Over the last decades, a rapid increase in the prevalence of obesity arising from high caloric diet intake and sedentary lifestyle is driving a global pandemic of T2D. Currently, 415 million people (about 9% of whole adult population) across the world have T2D. During the next decade, the number of T2D patients is expected to rise to around 642 million persons [2]. Obviously, genetics plays a crucial role in driving this disease; however, the dramatic increase in T2D incidence across the globe cannot be explained by genetic factors alone but must involve environmental factors as well [3]. There is increasing experimental and epidemiological evidence that the risk of development of T2D can be influenced not only by actual adult-life environmental conditions (primarily, lifestyle ones) but also by conditions in early life [3]. Convincing evidence that risk of T2D cannot be completely attributable to genetic predisposition and/or adult-life environmental factors was obtained, e.g., in a study on Pima Indian nuclear families in which at least one sibling was born before and other after the mother was diagnosed with T2D [4]. In this research, those siblings conceived after the mother has been diagnosed with T2D were 3.7 times more likely to have T2D compared to siblings born before their mother developed diabetes, even though they lived in similar conditions the rest of their life.

In the present review, we have summarized and discussed findings on this topic from epidemiological studies conducted with quasi-experimental design (‘natural experiments’).

## 2. Conceptual Framework for Developmental Nutritional Programming of Type 2 Diabetes (T2D)

According to the developmental programming of health and disease (DOHaD) hypothesis, which has been confirmed by many research findings over the past decades, the physiology and structure of the developing organism may be adapted in response to unfavourable environmental conditions, thereby predisposing it to many pathological conditions in adult life [5]. In particular, poor nutritional environments in early life can induce structural and functional changes in key organs responsible for nutrient regulation, including brain, liver, adipose tissue, muscle and pancreas [6]. Presently, this view is commonly referred to as the ‘predictive adaptive response (PAR)’ concept [7]. Exposure to adverse environmental factors such as inadequate or unbalanced nutrient supply during in utero development may ‘program’ for the long term appetite regulation, feeding behaviour, as well as adipose tissue and pancreatic beta cell dysfunction in the developing foetus [3]. As a result of these processes, the foetus may be adapted to adverse nutritional conditions by reducing ability to produce insulin and by occurrence of insulin resistance. According to the ‘thrifty phenotype’ hypothesis [8], such metabolic adaptation may provide short-term survival benefit in a poor postnatal environment via enhanced capacity to store fat in conditions of irregular availability of food resources, but may predispose the child to T2D development in conditions of food abundance in postnatal life. More specifically, in malnourished conditions when the foetus exhibits poor growth in utero (commonly referred to as intrauterine growth restriction, IUGR), the foetal adaptation to undernutrition is realized by a variety of mechanisms responsible for the energy and glucose metabolism, such as enhanced peripheral insulin sensitivity for glucose utilization, increased hepatic glucose production, lowered insulin sensitivity for protein synthesis in muscle, and impaired pancreatic development [9]. All these mechanisms provide obvious survival benefit for the IUGR foetuses by promoting both energy uptake and utilization, reducing the demand for amino acids and anabolic hormone production, and elevating glucose production to maintain glucose supply to vital organs, primarily the heart and brain. These adaptations lead to asymmetrical growth restriction of the foetus. The muscle and subcutaneous tissues exhibit the most pronounced growth restriction, while the least pronounced growth restriction is peculiar to the growing brain. Collectively, such adaptations allow IUGR foetal tissues to maintain the energy-dependent basal metabolic functions at the expense of body growth in conditions of reduced nutrient supply. If these adaptive modifications persist, or are more readily inducible later in life, they have the potential to promote energy absorption beyond metabolic capability when energy supplies increase, thereby causing insulin resistance, obesity and T2D in adulthood [9]. Among the factors affecting the risk of metabolic dysfunctions, including T2D, in adulthood, the prenatal and early postnatal malnutrition (both under- and overnutrition) is currently believed to be most important [10,11]. It should be noted that in this review only one aspect of malnutrition i.e., undernutrition but not overnutrition will be discussed.

The majority of early population studies used birth weight as a proxy for foetal conditions. From the data obtained, it has been initially concluded that low birth weight is a risk factor for T2D and that birth weight is inversely related to the disease risk [12]. In addition to T2D, low birth weight is a predictor of other T2D-associated conditions and complications later in life, including the impaired body composition and fat distribution [13], fasting lipid profile, blood pressure and insulin resistance [14], life-long activation of the hypothalamic-pituitary-adrenal axis [15], as well as coronary heart disease in adulthood [16]. Several more recent studies, however, found that a relationship between birth weight and risk of T2D is not linear but rather U-shaped, and high birth weight (>4000 g) is associated with an increased risk of T2D to the same extent as low birth weight (<2500 g) [17]. 

An association between low birth weight and risk of T2D in later life is most thoroughly studied to date. This association is apparently mediated by catch-up growth early in life which is an important risk factor for later T2D. The catch-up growth leads to a disproportionately enhanced rate of fat gain in comparison with lean tissue gain [18]. Such preferential catch-up fat is partly driven by mechanisms of energy conservation operating through suppression of thermogenesis and resulting in the development of thrifty ‘catch-up fat’ phenotype generally characterized by insulin and leptin resistance. Abnormalities in the growth hormone/insulin-like growth factor-1 (GH/IGF-1) axis, known to play a central role in promoting human growth and development, have been repeatedly reported in children born small for gestational age (SGA) [19]. Such long-lasting abnormalities of IGF-1 in SGA children with catch-up growth are believed to be critically implicated in the association with metabolic disorders, including T2D, later in life. 

Precise molecular mechanisms responsible for the nutritional developmental programming of T2D are not yet thoroughly characterized. In many recent studies, compelling evidence was provided that changes in epigenetic regulation of gene expression (heritable alterations in gene function without changes in the nucleotide sequence) is the most plausible mechanism for the link between unfavourable conditions in early development and adverse health outcomes in later life [20]. The main epigenetic mechanisms are DNA methylation and post-translational modifications of histone tails, as well as regulation by non-coding RNAs (microRNAs and long non-coding RNAs) [21]. Evidence for the key role of DNA methylation and other epigenetic mechanisms in mediating the risk of T2D and obesity has been repeatedly documented over the past years [22]. Initial evidence for the role of epigenetic regulation in obesity and T2D has been mainly provided by studies in animal models. These studies reported changes in epigenetic marks in key metabolic tissues following feeding with high-fat diet and by human investigation that demonstrated epigenetic alterations in T2D and obesity candidate genes in obese and/or diabetic persons. More recently, rapid technological advances and price reduction in epigenetic methodologies led to a rapid expansion of epigenome-wide association studies (EWAS) in human epidemiological examinations [22]. These studies clearly demonstrated epigenetic differences between diabetic and healthy control individuals, as well as epigenetic alterations associated with lifestyle interventions.

Within the DOHaD concept, an important point is that throughout embryonic and foetal development, intense epigenetic remodelling takes place that is necessary for the establishment of transcriptional programs responsible for cellular proliferation and differentiation. During these sensitive developmental periods, the epigenome is especially plastic and most sensitive to environmental disturbances [23]. Numerous research findings suggest that early-life adverse events (i.e., insufficient nutrition in utero) might be epigenetically ‘imprinted’ and ‘remembered’ decades later, thereby permanently influencing the metabolic phenotype [24]. There is convincing evidence that epigenetic alterations, including those triggered by early-life events and persisting through adulthood, is an important etiological factor in the development of T2D. Changes in DNA methylation and associated changes in patterns of expression of genes implicated in various aspects of glucose metabolism such as β-cell dysfunction, glucose intolerance and insulin resistance, have been shown to be critically involved in the pathogenesis of T2D [25]. The specific DNA methylation markers have been repeatedly identified in peripheral blood and pancreatic islets of the T2D patients (for review, see [26]).

**Figure 1:**
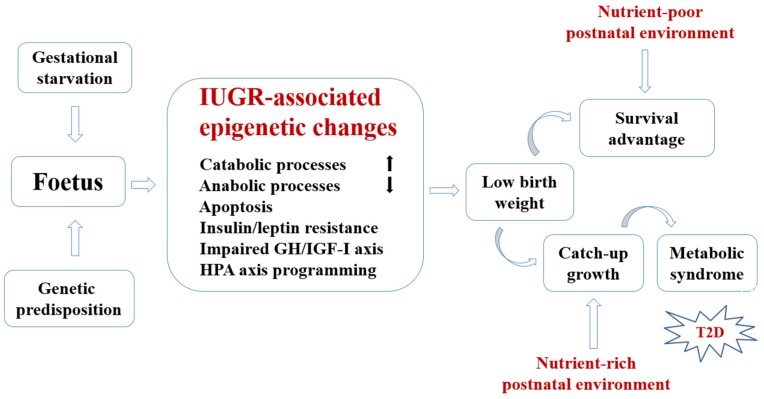
Schematic representation of hypothetical regulatory pathways responsible for developmental programming of type 2 diabetes (T2D) through prenatal undernutrition followed by catch-up growth in a nutrient-rich postnatal environment.

A schematic representation of hypothetical regulatory pathways responsible for developmental nutritional programming of T2D is presented in Figure 1.

## 3. Evidence from Animal Models

The bulk of evidence linking intrauterine and/or early postnatal nutrient environment and predisposition to beta-cell failure and T2D in adulthood comes from animal models (for review, see [27,28,29]). Most of these studies have used rodent models of 50% maternal dietary restriction (DR) during pregnancy to examine the postnatal beta-cell mass development in pups exposed to either normal or restricted post-natal nutritional conditions. Rodents exposed to intrauterine DR and subsequent normal or restricted post-natal nutrition exhibited diminished beta-cell mass both at birth (30%–50% vs. control) and throughout the early postnatal development (50%–70% vs. control) [30,31,32,33]. In adulthood, these animals were unable to adaptively enhance beta-cell mass in response to rising metabolic demand and consequent insulin resistance. As a result, they developed diabetic phenotypes characterized by beta-cell failure due to insufficient expansion of beta-cell mass, impaired insulin secretion, glucose intolerance and fasting hyperglycaemia [34,35]. For example, in a study using cross-fostering methodology to isolate effects of selective pre- and postnatal 50% DR [36,37], prenatal DR resulted in a ~50% reduction in beta-cell mass whereas postnatal DR led to decreased body weight, but both beta-cell mass and beta-cell fractional area were increased compared with control animals. These findings indicate that prenatal DR largely determines endocrine cell development while postnatal DR primarily impacts development of the exocrine pancreas [37].

Currently, molecular mechanisms responsible for impaired formation of beta-cell mass in response to early-life DR have come under intensive investigation. Among them, mechanisms of epigenetic regulation of gene activity seem to play a dominant role [38,39,40]. Inadequate nutritional environment during intrauterine development suppressed transcription of key genes regulating beta-cell development in rats [39]. Feeding pregnant females with a low-protein diet led to hypomethylation of genes encoding glucocorticoid receptor and peroxisome proliferator-activated receptor gamma in the offspring livers. In a rat model, maternal DR also resulted in a significant reduction in the levels of expression of genes encoding key transcription factors regulating embryonic beta-cell development such as the pancreatic and duodenal homeobox 1 (PDX-1) [41,42]. Such changes on the epigenetic level were accompanied by reduced postnatal beta-cell formation and incapability to expand beta-cell mass in response to metabolic stress. Moreover, maternal DR diminished the postnatal expression of *Pdx-1* gene in pancreatic exocrine ducts which is suspected to harbor a putative pool of pancreatic beta-cell progenitor population in adult rodents [39]. Other factors potentially contributing to these effects are hormones that operate during foetal life, such as insulin, insulin-like growth factors, glucocorticoids, as well as some specific molecules such as taurine [43].

In several studies, maternal protein restriction has been shown to program an insulin-resistant phenotype in rodents, especially in consequence of catch-up growth following intrauterine growth restriction. Such mode of malnutrition resulted in expression of early markers of insulin resistance and metabolic disease risk, including alterations in adipocyte cell size and expression levels of several insulin-signalling proteins through post-transcriptional mechanisms [44]. Catch-up growth following maternal protein restriction also favoured the development of obesity in adult male rat offspring [45]. In a mice model, a protein restriction during foetal life followed by catch-up growth led to obesity in adult male mice [46]. These changes were associated with increased relative fat mass, hypercholesterolemia, hyperglycaemia and hyperleptinemia, and also with altered expression profile of several gene-encoding enzymes involved in lipid metabolism. 

## 4. Quasi-Experimental Design in Studying the Developmental Origin of T2D

The experimental research of developmental programming in human populations is not applicable, either for ethical reasons and because the long-term follow-up is required to observe life-long outcomes of early-life experiences. In this regard, an important point is that observational studies in appropriate populations may be realized. The consistent evidence linking the early-life conditions with adult health status has been accumulated from studies conducted with a quasi-experimental design (‘natural experiments’), defined as “naturally occurring circumstances in which subsets of the population have different levels of exposure to a supposed causal factor, in a situation resembling an actual experiment where human subjects would be randomly allocated to groups” [47]. Both natural and man-made disasters such as famine obviously provide a lot of advantages to their use in quasi-experimental studies. In the sections below, empirical findings from such a line of research across countries are reviewed.

### 4.1. Dutch Famine of 1944–1945

The long-term health consequences of the Dutch famine (‘Hunger Winter’) are the most comprehensively studied up to now. This famine, caused by the Nazi food embargo, affected the western Netherlands from November 1944 to May 1945. Many features of the Dutch famine can be used in a quasi-experimental design. It was a severe famine, distinctly defined in time and place and occurred in a society with a well-developed structure of administrative control. Therefore, exposure to this famine may be accurately defined by region and date of birth in relation to distribution of the food rations and the level of calories consumed. Such circumstances of the Hunger Winter famine provide the opportunity to thoroughly examine the link between inadequate maternal nutrition during particular trimesters of pregnancy and the offspring’s adult health status. While a normal daily ration is 2000 kcal and 2500 kcal for women and men, respectively, the average daily rations during the famine were less than 700 kcal [48]. The population that suffered from severe food shortage throughout the famine was generally well fed before and after this period. These features of the Dutch famine provide the researchers with a near-ideal quasi-experimental research design to examine how maternal malnutrition throughout the critical early-life time windows can affect the life-course offspring health status. The prenatal exposure to the Dutch famine has been repeatedly shown to be related to the impaired metabolic phenotypes such as elevated levels of plasma lipids and body mass index (BMI), as well as enhanced risks of obesity and cardiovascular disease (CVD) later in life (for reviews, see refs. [48,49,50]). Most of these associations have been critically dependent on the timing of exposure. In the majority of the studies, early gestation was found to be the most vulnerable period [48,49,50,51]. Childhood and puberty are other sensitive periods with high potential to trigger programming effects. The link between exposure to the Dutch famine between age 0 and 21 years and T2D in adulthood was clearly evident, e.g., from the study by van Abeelen et al. [52]. This relationship was found to be dose-dependent: in those women who self-reported moderate famine exposure during their childhood and young adulthood, the age-adjusted hazard ratio for T2D was 1.36, and in those who reported severe famine exposure, the hazard ratio was 1.64 compared to unexposed women. The exposure to severe malnutrition during the Dutch famine at ages 11−14 was found to be considerably associated with enhanced probability of developing T2D and/or peripheral arterial diseases at ages 60−76 in women, but not in men [53].

In the Dutch famine study, compelling evidence has been obtained that exposure to famine during prenatal development may result in persistent epigenetic changes. Although no relationship between the prenatal exposure to the Dutch famine and overall global DNA methylation in adulthood was observed [54], levels of methylation of particular genes were clearly associated with prenatal famine exposure. The methylation levels of the imprinted gene encoding an insulin-like growth factor 2 (IGF2), known to play a crucial role in human growth and development, have been estimated by Heijmans et al. [51]. This gene was selected for analysis because its methylation marks are stable up to adult age, making *IGF2* gene a good candidate for such a study. In this research, those subjects exposed to the Dutch famine during their early gestation period had much lower *IGF2* methylation levels compared to control unexposed individuals six decades after the hunger exposure. Subsequently, this observation has been extended by examination of a set of 15 additional candidate loci responsible for development of metabolic and cardiovascular phenotypes [55]. Levels of methylation of six of these loci (*GNASAS*, *IL10*, *LEP*, *ABCA1*, *INSIGF* and *MEG3*) have been found to be associated with prenatal exposure to famine. 

### 4.2. Famines in 20th-Century Austria 

Findings from the Dutch Hunger Winter Study on the developmental origin of T2D were also confirmed in populations of other countries such as Austria, which has been subjected to three massive famine episodes during the 20th century. These famines occurred in 1918–19 during the collapse of the Austro-Hungarian Empire; in 1938, following the economic crisis, harvest failure, and food embargo from Nazi Germany; and in 1946–1947 in the period following the Second World War. Based on the data set including 325,000 Austrian diabetic patients, Thurner et al. [56] observed an excess risk of T2D in those persons who were born during or immediately after the periods of these famine episodes. For instance, up to 40% higher chances of having T2D in those individuals who were born in 1919–1921 compared to those who were born in 1918 or 1922, have been revealed in different Austrian regions. Noteworthy, the excess risk of T2D was practically absent in those Austrian provinces that were less affected by hunger. Furthermore, T2D rates have been correlated with the economic wealth of particular regions. The authors concluded that the revealed peaks of T2D in subjects born during and after the periods of severe starvation obviously demonstrate importance of environmental determinants in the period from conception to early childhood, in addition to genetic predisposition and shared life-course factors. These determinants clearly include nutritional triggers, although contribution of other triggering factors such as the famine-related stress and infectious factors, including rodent-borne viral infections, cannot be excluded [57]. The data obtained from this research, however, collectively favoured the hunger hypothesis as the leading explanation for the effects observed [56,58].

### 4.3. Ukrainian Famine of 1932–1933 

The association between prenatal exposure to the famine and adult risk of T2D has been recently examined in large birth cohorts (total *n* = 43,150) born before, during and after the Great Ukrainian Famine of 1932–1933 (‘Holodomor’) [59]. This famine was caused by the Soviet Union government’s forced agriculture collectivization throughout the early 1930s and led to the deaths from starvation of several million people with a ten-fold increase of mortality rate in April−July 1933 compared to the pre- and post-famine times. The cohorts born during the famine can be well defined with respect to the timing of the famine exposure in relation to the stage of pregnancy and the severity of the famine around the birth date. The odds ratios (ORs) for developing T2D were 1.47 in those individuals born in the first half of 1934 in regions affected by extreme famine, 1.26 in those born in regions with severe famine, and there was no increase (OR = 1.0) in those born in regions with no famine, compared to the births in other examined time periods. The associations observed between T2D and famine exposure around the time of birth have been found to be similar in men and women. The data obtained showed a dose-response relationship between the famine severity during the prenatal development and the risk of T2D later in life, and assumed that early gestational stage is a critical time window for modulating the prenatal environment to affect the adult T2D risk.

### 4.4. Leningrad Siege of 1941–1944

The Nazi Siege of the Russian city of Leningrad (the modern-day St. Petersburg) in 1941–1944 resulted in extreme hunger and death of about a million city residents. The siege-induced starvation caused an average fall in birth weight of 500–600 g [60]. Follow-up of 549 subjects born in Leningrad before or during the siege, however, demonstrated no effect of intrauterine undernutrition during the siege on dyslipidaemia, glucose intolerance, hypertension and the risk of CVD in adulthood [61,62]. Starvation-exposed individuals demonstrated only evidence for endothelial dysfunction and for a stronger influence of obesity on blood pressure. These results seem to contradict the thrifty phenotype concept since in utero undernutrition was not related to glucose intolerance in adult persons, although prenatal malnutrition influenced their blood pressure and differ from those obtained in the Dutch Hunger Winter study. One possible explanation for such a contradiction suggested by some authors in discussing these data is that the Leningrad siege research was complicated by the fact that malnutrition extended into the postnatal period. Thus, the conflict between prenatal and postnatal environments did not occur [63]. Indeed, in the Netherlands, the food supplies become fully adequate after the war ended, while those babies who were born throughout the Leningrad siege remained malnourished during all their childhood, since this famine lasted for years rather than months and nutrition was poor in subsequent years as well. The association between starvation during the Leningrad siege in early life and the risk of T2D development in adulthood was, however, observed in several more recent studies. Both the increasing incidence and decreasing age of onset of T2D without obesity have been found in women exposed to starvation throughout the Siege of Leningrad during their childhood [64]. This cohort was characterized by higher incidence of conditions associated with metabolic dysregulation such as severe arterial hypertension, and also atherosclerosis of coronary, brain and carotid arteries [65]. Similar health problems were also demonstrated in cohorts exposed to starvation during the Leningrad Siege in their childhood and puberty. Women who were 6−8 years old and men who were 9−15 years old throughout the peak of the famine demonstrated higher systolic blood pressure in their adulthood as compared with unexposed individuals who were born during the same period. Moreover, men exposed to hunger at age 6−8 and 9−15 were characterized by increased mortality from ischaemic heart disease and cerebrovascular disease, respectively [66].

### 4.5. Chinese Famine of 1959–1961

The long-term health consequences of the Chinese Famine of 1959–1961 (‘Great Leap Forward Famine’) are extensively studied now. This massive famine occurred in China in the late 1950s following the disastrous social agricultural reform commonly referred to as ‘Great Leap Forward.’ Over the years of the famine, 25 to 30 million more deaths and 30 to 35 million fewer births were registered in China than would have been expected under normal conditions [67]. In recent years, the Great Leap Forward Famine is the most actively studied famine episode across the globe. It should be noted, however, that one methodological limitation of the Chinese Famine study is that famine exposure data are not available by month; therefore, the periods of the famine exposure cannot be as precisely defined as in the Dutch famine study or in the Ukrainian study.

In most of the studies of long-term impacts of the Chinese Famine, the evidence was obtained that T2D as well as associated metabolic abnormalities were more common among adult Chinese residents born during the famine than among control individuals born after the famine (for a systematic review, see [68]). More specifically, in areas which were severely affected by famine, those subjects who were exposed to famine prenatally had a 3.9-fold enhanced risk of hyperglycaemia in comparison with non-exposed individuals; this difference was not seen in less severely affected regions. Remarkably, the hyperglycaemia risk was 7.6-fold higher in those prenatally exposed subjects who followed an affluent/Western dietary pattern and 6.2-fold higher in those who had a higher economic status in later life compared to non-exposed controls [69]. In a more recent study by Wang et al. [70], both prenatal and childhood exposures to famine were shown to result in higher risk of being diagnosed with T2D in adulthood (1.5-times and 1.8-times, respectively), compared with non-exposed subjects. Individuals residing in Chinese regions with high economic status had a greater T2D risk (OR = 1.46). Interestingly, the timing of association between the famine exposure in early life and adult T2D was gender-specific: an elevated risk of T2D development was evident in the foetal-exposed men (OR = 1.64) and childhood-exposed women (OR = 2.81). These findings were further confirmed in subsequent research by the same authors, where a significant association between the famine severity in the areas of exposure and the risk of T2D was found [71]. Those subjects who were exposed to severe famine during the foetal and childhood periods had substantially higher odds estimates (1.90 and 1.44, respectively). A significant interaction between the level of famine severity in the areas of exposure throughout the prenatal and childhood periods and the risk of T2D in adulthood has been observed. In another Chinese population, 1.44-fold higher risk of T2D development in the middle-childhood-exposed group, and 1.5-fold higher risks of hyperglycaemia in both the middle- and late-childhood-exposed groups were demonstrated compared to the unexposed group [72]. Remarkably, those individuals who experienced more severe famine in childhood had a 38% higher risk of T2D development than those exposed to less severe famine. The revealed association was, however, sex-specific and has been found in women, but not in men. Similar associations have been observed for the hyperglycaemia risk as well. 

In a recent study conducted in Suihua, China, the evidence was obtained that programming effects can be manifested not only in those prenatally exposed to famine population (F1 generation), but also in the F2 progeny [73]. In this research, prenatal exposure to the Chinese Famine has been linked to a 1.75-fold enhanced risk of T2D and 1.93-fold enhanced risk of hyperglycaemia in F1 adult offspring in comparison with unexposed individuals. Furthermore, F2 offspring of exposed ancestors had a 2.02-fold elevated risk of adult hyperglycaemia compared to the offspring of non-exposed ancestors. These findings suggest that famine-induced effects can be transmitted via the germ line across generations and translated into increased T2D susceptibility in the descendants of the famine-exposed individuals.

### 4.6. Nigerian Famine of 1967–1970

The Nigerian Famine (commonly referred to as ‘Biafran Famine’) occurred during the Nigerian Civil War from 1967–1970. Of the one to three million Nigerians that died during this civil war, only a relatively small fraction (about 10%) had lost their lives from military action as such; the majority died from war-associated starvation [74]. The risks of glucose intolerance, hypertension and being overweight 40 years after prenatal exposure to the Biafran Famine have been assessed in the Hult et al. study [75]. The studied cohorts (total *n* = 1339) included those adults born before (1965−1967), during (1968−1970), or after (1971−1973) the years of famine. The exposure to famine during both foetal and infant periods has been found to be associated with significantly increased systolic and diastolic blood pressure, higher levels of p-glucose and waist circumference, as well as with substantially elevated risks of systolic hypertension (OR = 2.87), impaired glucose regulation (OR = 1.65) and overweight (OR = 1.41) in adulthood compared with persons who were born after the famine. As in the case of the Chinese Famine study, the lack of birth weight data and the resulting impossibility to separate effects of prenatal and infant famine exposure is the main methodological weakness of Biafran Famine research.

### 4.7. Holocaust (1939−1945)

The Holocaust was a genocide in which Nazi Germany and its collaborators killed about six million Jews. It was obviously associated with severe starvation and stress in affected populations. The long-term health outcomes of exposure to the Holocaust in the period from preconception to early infancy were determined in recent studies conducted in Israel. The pilot study involved 70 European Jews born in countries under Nazi rule during the period 1940−1945 (exposed group) and 230 age- and sex-matched Israeli-born individuals (non-exposed group) who self-reported the presence of chronic diseases [76]. The exposed individuals have been shown to be at a higher risk of adult metabolic disturbances, including enhanced BMI, as well as 1.46-fold increased risk of hypertension, 1.58-fold increased risk for dyslipidaemia, and 1.89-fold increased risk of T2D compared to the Holocaust-unexposed group. The associations observed were further confirmed on larger groups of participants (exposed group, *n* = 653; non-exposed group, *n* = 433) [77]. The higher risks of hypertension (OR = 1.52), T2D (OR = 1.60), metabolic syndrome (OR = 2.14) and vascular disease (OR = 1.99) were found in exposed individuals.

In general, findings from quasi-experimental studies suggest that exposure to famine in early life may result in serious metabolic disturbances in later life including high risk of development of T2D and associated conditions. The main findings from these studies are summarized in Table 1. 

## 5. Seasonality of Birth

Season of birth also can be used in quasi-experimental design to examine associations between early-life exposures, including nutritional ones, and later-life health outcomes. In this regard, month of birth presents a good instrument which may help to examine later-life outcomes of early-life exposures independently of life-course factors. This is true since decades ago there were strong seasonal variations in nutrition, especially in developing countries. Availability of cereals, vegetables, fruits and animal proteins varied significantly according to the season. Such differences in the supply of high-quality food might potentially affect the foetal and neonatal development depending on the month of gestation [89]. Other potentially confounding factors for early-life disease programming, including temperature [90], infections [91], sunlight/photoperiod and, correspondingly, production of melatonin and vitamin D [92], as well as maternal lifestyle factors such as physical activity [93] and alcohol intake [94], also tend to vary seasonally. 

Seasonal conditions around the period of birth were demonstrated to significantly determine birth weight: lower birth weights were observed in the winter-born newborns and higher birth weights in summer-born newborns in the high- and low-latitude areas, while the summer birth was associated with relatively lower birth weight in the mid-latitude areas [95]. Seasonality of birth has been demonstrated for many aspects of metabolic syndrome, including high systolic blood pressure [82], obesity [83,84] and also dyslipidaemia, insulin resistance and CVD [85]. The seasonal pattern of birth for childhood autoimmune (type 1) diabetic patients was reported repeatedly (see, e.g., [96]), while the seasonality of birth for T2D adult persons was observed only in a few studies. Seasonal patterns of birth were reported, e.g., in small-sample studies conducted in 155 adolescent African-Americans [86] and in 282 T2D patients in the Netherlands [87].

By now, the most obvious evidence for the seasonality of birth in T2D patients is provided in research conducted in a Ukrainian population [88]. In this study, those persons who were born in April−May had increased risk ofT2D development. In the climatic conditions characteristic of the Ukraine, these subjects ordinarily experienced their foetal life in the nutritionally marginal period from late autumn to early spring and passed the first neonatal months during the relatively plentiful season. In contrast, a decreased risk of T2D was observed in those born in November–December. In these individuals, prenatal development in a nutritionally abundant season would have been followed by early infancy in the season of relative scarcity (winter−spring). The first scenario is apparently more high-risk for developing T2D than the second one. These results are highly consistent with the thrifty phenotype hypothesis [8]. Interestingly, the seasonal pattern of birth was found to be very similar in type 1 and type 2 diabetic patients, suggesting shared early-life etiological causation for both disorders [97]. In more recent research by Jensen et al. [98], no evidence for seasonality of birth in Danish patients with T2D was found. The authors assumed that difference in effects obtained in Denmark and the Ukraine may be explained by standards of living or by differences in latitude between the countries. Indeed, as the seasonal variations in both weather and nutrition were much more pronounced in the Ukraine than in Denmark throughout the study periods, and since the Ukraine belonged to low-income countries for much of that time, its residents experienced more pronounced seasonal extremes than those experienced by people in a more prosperous country like Denmark. 

In discussing the mechanistic basis for seasonal programming of adult-life diseases, one possible explanation is that seasonal factors operating around the time of birth may trigger persistent epigenetic changes that have adaptive significance in postnatal development but can predispose to chronic disorders, including the metabolic ones, at the late stages of life. The evidence for the link between season of birth and long-term changes in DNA methylation has been recently obtained in the epigenome-wide association study (EWAS) by Lockett et al. [99], where methylation at 92 CpG dinucleotides was significantly associated with season of birth. The networks related to the cell cycle, development and apoptosis have been found to be enriched among these differentially methylated CpG sites. Interestingly, the season-associated methylation patterns have been mainly absent in newborns, suggesting they arise postnatally. Although these findings were not confirmed in a more recent study by Dugué et al. [100], they, however, suggest that changes in DNA methylation might mechanistically underlie the season-of-birth effects on the risk of later-life disease.

## 6. Conclusions and Future Perspectives

A trend to a dramatic enhancing incidence of type 2 diabetes (T2D) has become a serious problem across the globe over the past years. Metabolic syndrome and associated risk factors including dyslipidaemia, high blood pressure, impaired glucose metabolism and T2D, are among the main causes of death in both developed and developing countries. It is widely believed that risk of T2D is mostly dependent on genetic and lifestyle factors. However, while genetic factors undoubtedly contribute to an individual susceptibility to development of obesity and T2D, the identified genetic variants can explain only part of the variation [22,101]. Recent research has demonstrated that exposure to unfavourable environmental stimuli early in life is another important determinant of the risk of T2D and associated conditions during adulthood. Findings from several of these studies suggest that epigenetic regulation can be largely contributed to development of these pathological states. Since epigenetic marks may persist long term, epigenetic modifications triggered by environmental cues throughout early sensitive stages may lead to lasting effects on the metabolic functioning, thereby affecting the risk of metabolic disorders, including T2D, later in life [102]. Prenatal and early postnatal nutrition is likely the most important factor affecting the adult risk of T2D. For instance, in studying the long-term health consequences of prenatal exposure to the Dutch famine, a link between poor nutritional intake in utero and impaired glucose regulation, atherogenic lipid profiles and obesity later in life, all known to be risk factors for development of T2D, has been demonstrated [99,100,101,102]. Therefore, it is not surprising that cohorts exposed to starvation in early life are at higher risk of T2D development. In a very recent meta-analysis of 11 published articles, a strong association between exposure to famine in early life and increased risk of T2D in adulthood has been observed (the pooled relative risk (RR) = 1.38, 95% CI 1.17−1.63) [103]. RRs for T2D development were 1.36 (95% CI 1.12−1.65) for cohorts exposed prenatally or during the early postnatal period and 1.40 (95% CI 0.98−1.99) for those cohorts who were exposed in their childhood compared to the unexposed cohorts.

In offspring born to mothers experiencing famine during pregnancy, differential methylation of genes, including those associated with pathogenesis of T2D, has been observed [51,55], indicating the importance of epigenetic processes in mediating early-life starvation exposure to the risk of later-life disease. Data from reviewed studies suggest that a focus on very early periods of gestation, and perhaps even on the periconceptional period, should constitute the next frontier for prevention of T2D over the human life course [104]. Some studies have indicated that epigenetic effects contributing to development of T2D could be transmitted across several generations. In research conducted in the Överkalix, an isolated community in northern Sweden, the possibility of transgenerational effects on T2D mortality was observed. The transgenerational consequences of the ancestors’ nutrition throughout their slow growth period (SGP, aged 9 to 12 years), the period of higher susceptibility of organism to environmental influences, were investigated in cohorts born in this region in 1890, 1905 and 1920 [105,106]. In case of limited food availability in the father’s SGP, then the descendant cardiovascular mortality was low, while the overeating of paternal grandfathers led to a four-fold increase in diabetes mortality in the offspring [105,106]. Such transgenerational effects were shown to be gender-specific: the paternal grandmother’s nutrient supply affected granddaughters’ mortality risk, while the paternal grandfather’s nutrient supply was shown to be associated with the mortality risk in grandsons [106]. Since epigenetic alterations unlike genetic mutations are potentially reversible [107], pharmacological modification of epigenetic marks contributing to T2D development can provide a novel approach to prevention and treatment of T2D and associated disorders. 

## Figures and Tables

**Table 1:** Summary of main findings from research on long-term metabolic health consequences of early-life undernutrition exposure

Country	Cause of Starvation	Period	Adult consequence	Ref.
Netherlands (‘Dutch Hunger Winter’)	Nazi food embargo	1944–1945	Impaired glucose regulation Atherogenic lipid profiles Obesity, CVD T2D Lower *IGF2* methylation Changed methylation of *ABCA1*, *GNASAS*, *IL10*, *LEP*, *INSIGF* and *MEG3* genes	[78] [79,80,81] [49,81] [49] [51] [55]
Austria	Empire’s collapse Nazi food embargo Post-war period	1918–1919 1938 1946–1947	High risk of T2D	[56]
Ukraine (‘Holodomor’)	Agriculture collectivization	1932–1933	High risk of T2D	[59]
Russia	Leningrad Siege	1941–1944	Endothelial dysfunction, stronger influence of obesity on blood pressure Increasing incidence of T2D Atherosclerosis, arterial hypertension	[61,62] [64] [65,66]
China (‘Great Leap Forward Famine’)	Disastrous social agricultural reform	1959–1961	Hyperglycemia High risk of T2D	[69,73] [70,71,72,73]
Nigeria (‘Biafran Famine’)	Civil war	1967–1970	Increased blood pressure, higher levels of p-glucose, increased waist circumference, overweight, high risks of impaired glucose regulation and systolic hypertension	[75]
Europe (‘Holocaust’)	Nazi genocide	1939–1945	Enhanced BMI, hypertension, dyslipidemia, high risk of T2D and CVD	[76,77]
Spain	Seasonal malnutrition	1935−1954	High systolic blood pressure	[82]
United Kingdom	Seasonal malnutrition	1920–1930	Obesity	[83]
Canada	Seasonal malnutrition	1943–1995	Obesity	[84]
United Kingdom	Seasonal malnutrition	1924–1943	Dyslipidaemia, insulin resistance and CVD	[85]
USA	Seasonal malnutrition	1968–1995	High risk of T2D	[86]
Netherlands	Seasonal malnutrition	1920–1948	High risk of T2D	[87]
Ukraine	Seasonal malnutrition	1930–1938	High risk of T2D	[88]
